# CircRNA Is a Rising Star in Researches of Ocular Diseases

**DOI:** 10.3389/fcell.2020.00850

**Published:** 2020-09-03

**Authors:** Chengshou Zhang, Jianghua Hu, Yibo Yu

**Affiliations:** ^1^Eye Center of the Second Affiliated Hospital, School of Medicine, Zhejiang University, Hangzhou, China; ^2^Department of Ophthalmology, Jiande Branch, The Second Affiliated Hospital, School of Medicine, Zhejiang University, Hangzhou, China

**Keywords:** circular RNA, noncoding RNA, microRNA sponge, ocular diseases, ophthalmology

## Abstract

A newly rediscovered subclass of noncoding RNAs, circular RNAs (circRNAs), is produced by a back-splicing mechanism with a covalently closed loop structure. They not only serve as the sponge for microRNAs (miRNAs) and proteins but also regulate gene expression and epigenetic modification, translate into peptides, and generate pseudogenes. Dysregulation of circRNA expression has opened a new chapter in the etiology of various human disorders, including cancer and cardiovascular, neurodegenerative, and ocular diseases. Recent studies recognized the vital roles that circRNAs played in the pathogenesis of various eye diseases, highlighting circRNAs as promising biomarkers for diagnosis and assessment of progression and prognosis. Interventions targeting circRNAs provide insights for developing novel treatments for these ocular diseases. This review summarizes our current perception of the properties, biogenesis, and functions of circRNAs and the development of circRNA researches related to ophthalmologic diseases, including diabetic retinopathy, age-related macular degeneration, retinopathy of prematurity, glaucoma, corneal neovascularization, cataract, pterygium, proliferative vitreoretinopathy, retinoblastoma, and ocular melanoma.

## Introduction

Noncoding RNAs (ncRNAs), containing microRNAs (miRNAs) and long noncoding RNAs (lncRNAs) comparatively studied extensively, are identified to have vital functions in the regulation of gene expression and development of many human diseases (Esteller, 2011). A rising star in studies of endogenous ncRNAs is circular RNA (circRNA), which has a single-stranded covalently closed structure without 5′ caps and 3′ poly-A tails (Lasda and Parker, 2014) and has attracted interest from many researchers. Although circRNAs were initially discovered in plant viroids four decades ago (Sanger et al., 1976) and then sporadically in hepatitis delta virus and transcripts of the DCC gene, SRY gene and cytochrome P450 2C24 gene (Patop et al., 2019), these molecules were not well understood and treated as by-products of aberrant RNA splicing because they appeared in low abundance and had unknown biological functions (Cocquerelle et al., 1993). However, due to advances in bioinformatics tools and RNA high-throughput sequencing technology, circRNAs have been confirmed to exist in high abundance and be highly diverse and conserved. It has also been confirmed that they are widely expressed in eukaryotes with a cell/tissue- and developmental stage-specific manner (Salzman et al., 2012, 2013; Jeck et al., 2013; Memczak et al., 2013). Since the circRNA called CDR1as/ciRS-7 has been first reported to function as the sponge for miR-7, circRNAs are becoming the focus of biomedical studies (Hansen et al., 2013a; Memczak et al., 2013). Their unique properties and mysterious functions are being uncovered gradually. For instance, it has been found that, in addition to acting as miRNA sponges, circRNAs can interact with RNA-binding proteins (RBPs) (Ashwal-Fluss et al., 2014; Westholm et al., 2014; Conn et al., 2015; Ivanov et al., 2015), regulate RNA splicing or transcription (Ashwal-Fluss et al., 2014; Li Z. et al., 2015), and translate into peptides or proteins (Legnini et al., 2017; Pamudurti et al., 2017; Yang et al., 2017). CircRNAs are also implicated in various biological processes, such as cell differentiation, proliferation, migration and death, carcinogenesis, angiogenesis, neuronal genesis, and innate immune responses (Guo et al., 2014; Lasda and Parker, 2014; Li X. et al., 2018). Furthermore, emerging studies have revealed that circRNAs play a vital role in the pathogenesis of various diseases, including cancer (Su et al., 2019), cardiovascular disease (Aufiero et al., 2019), neurodegenerative diseases (Kumar et al., 2017), and ocular diseases (Guo et al., 2019). Thus, circRNAs can serve as novel biomarkers and therapeutic targets for many diseases.

**FIGURE 1:**
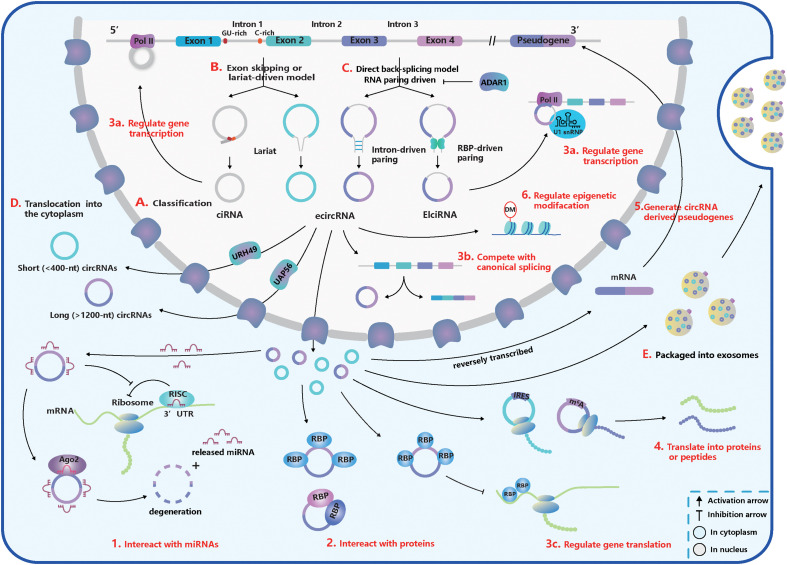
The basic acknowledgment of circular RNA (circRNA). **(A)** CircRNA classification. CircRNAs can be classified into intronic circRNA (ciRNA), exonic circRNA (ecircRNA), and exon-intron circRNA (ElciRNA). **(B)** Exon skipping or lariat-driven mechanism of circRNA biogenesis. Lariats contained skipping exons and introns, can produce ecircRNAs and EIciRNAs. And ciRNAs are produced from a lariat intron with special GU-rich element at 5′ splice sites (ss) and C-rich element at 3′ branchpoint site. **(C)** Direct back-splicing or RNA pairing-driven mechanism of circRNA biogenesis. Special elements in introns like inverted repetitive Alu elements and reverse complementary matches (RCMs) and special RNA-binding proteins (RBPs) like NF90/110, quaking (QKI), and muscleblind (MBL) can facilitate the RNA-pairing. But adenosine deaminase acting on RNA 1 (ADAR1) can inhibit the RNA-pairing and reduce circRNA expression. **(D)** The translocation of circRNAs from the nucleus into the cytoplasm. Short (<400 nt) and long (>1,200 nt) circRNAs were transported into the cytoplasm based on the URH49 and UAP56, respectively. **(E)** Packaged into exosomes. CircRNAs can execute the regulatory function by exporting exosomes. **(1–6)** The function of circRNAs. **(1)** Interact with miRNAs. CircRNAs can work as miRNA sponges and promote the translation of miRNA-targeted genes. But some miRNAs can trigger the circRNA degeneration in the Ago2-slicer-dependent manner, which leads to the release of binding miRNAs. **(2)** Interact with proteins. CircRNAs can serve as transporters, decoys, or scaffolds for proteins and regulate their activities. **(3a–c)** Regulate gene transcription, splicing, and translation. **(3a)** CircRNAs can inhibit gene translation by competing for special RBPs. **(3b)** CiRNA and EcliRNA can promote parent gene transcription by interacting with Pol II. **(3c)** CircRNA biogenesis competes with canonical splicing and circRNAs like circMbl can regulate gene splicing. **(4)** Translate into proteins or peptides. CircRNAs like circZNF609 have internal ribosome entry site (IRES) within their sequences and can be translated. CircRNA translation also can be driven by N^6^-methyladenosine (m^6^A) modification in a cap-independent manner. **(5)** Generate pseudogenes. **(6)** Regulate epigenetic alterations. CircRNAs can adjust epigenetic modifications, such as DNA or histone methylation.

In recent years, circRNA studies have begun to shed light on studies in the ophthalmology area. Dysregulated circRNAs exert a significant influence on the development of eye tissue and the pathogenesis of ocular diseases (George et al., 2019; Guo et al., 2019). However, to fully reveal the importance of circRNAs, greater knowledge is required, which can be obtained through further researches. Here, we present an overview of the characteristics, biogenesis, and functions of circRNAs (Figure 1) and their current recognition of and development for regulating ocular diseases (Table 1).

**TABLE 1:** Verified circRNAs involved in various ocular diseases.

Ocular diseases	circRNAs	Expression	Functions	Related networks	References
DR	circ_0005015	Upregulated in the plasma, vitreous samples, and FVMs of DR patients.	Promote the angiogenic function of HRVECs *in vitro*.	miR-519d-3p-MMP-2/STAT3/XIAP	Zhang S.-J. et al., 2017
	circHIPK3	Upregulated in HRVECs under HG conditions, DR models, the plasma, vitreous samples, and FVMs of DR patients.	Promote the angiogenic function of HRVECs under HG conditions *in vitro*. Aggravate the DM-induced retinal vascular dysfunction *in vivo*.	c-myb, miR-30a-3p/VEGFC/FZD4/WNT2	Shan et al., 2017
	circZNF609	Upregulated in HUVECs under HG conditions, DR models, the plasma, and FVMs of DR patients.	Promote the angiogenic function of HUVECs under HG conditions *in vitro*. Aggravate the DM-induced retinal vascular dysfunction *in vivo*.	miR-615-5p-MEF2A	Liu C. et al., 2017
	circDMNT3B	Downregulated in HRMECs under HG conditions and the FVMs of DR patients.	Suppress the angiogenic function of HRMECs under HG conditions *in vitro*. Ameliorate DM-induced retinal vascular dysfunction and visual damage *in vivo*.	miR-20b-5p-BAMBI	Zhu et al., 2019
	circPWWP2A	Upregulated in retinal pericytes under DM related stresses instead of HRVECs, DR models, and the FVMs of DR patients.	Promote the viability, proliferation, antiapoptosis, recruitment toward HRVECs of pericytes under DM-related stresses *in vitro* but affect HRVECs indirectly *via* exosomes. Ameliorate DM-induced retinal vascular dysfunction and pericyte dysfunction *in vivo*.	miR-579-angiopoietin1/occludin/SIRT1	Liu et al., 2019
	circZNF532	Upregulated in retinal pericytes following DM-related stresses, DR models, and the vitreous samples of DR patients.	Promote the viability, proliferation, antiapoptosis, recruitment toward HRVECs of pericytes under DM-related stresses *in vitro*. Ameliorate DM-induced retinal vascular dysfunction and pericyte dysfunction *in vivo*.	SP1, miR-29a-3p-CSPG4/LOXL2/CDK2	Jiang et al., 2020
ROP	circZNF609	Upregulated at the neovascularization stage of OIR models.	Aggravate the oxygen-induced retinal vascular dysfunction *in vivo*.	miR-615-5p-MEF2A	Liu C. et al., 2017
Exudative AMD	circZBTB44	Upregulated in chorioretinal ECs under hypoxic conditions, CNV models, the aqueous humor of nAMD patients.	Promote the angiogenic function of chorioretinal vascular ECs under hypoxic conditions *in vitro*. Aggravate the CNV development *in vivo*.	miR-578-VEGFA/VCAM1	Zhou et al., 2020
Atrophic AMD	circNR3C1	Downregulated in RPE cells under oxidative stress and the blood serum of AMD patients.	Maintain the RPE phenotypes and functions *in vitro* and *in vivo* to prevent AMD progression.	miR-382-5p-PTEN-AKT/mTOR	Chen X. et al., 2020
Glaucoma	circZRANB1	Upregulated in glaucoma model and the aqueous humor of glaucoma patients.	Promote the viability, proliferation, and activation of Müller cells directly, but indirectly inhibit RGC function *in vitro*. Promote the retinal reactive gliosis and glia cell activation (glaucomatous retinal neuropathy) *in vivo*.	miR-217-RUNX2	Wang et al., 2018b
	circZNF609	Upregulated in glaucoma model and the aqueous humor of glaucoma patients.	Promote the viability, proliferation, and activation of Müller cells directly, but indirectly inhibit RGC function *in vitro*. Promote the retinal reactive gliosis and glia cell activation (glaucomatous retinal neuropathy), but inhibit RGC survival *in vivo*.	miR-615-METRN	Wang et al., 2018a
ARC	circHIPK3	Downregulated in lens capsules of patients with various types of ARC.	Promote the viability, proliferation, EMT and antiapoptosis upon oxidative stress of HLECs *in vitro*.	miR-193a-3p-CRYAA	Liu et al., 2018, 3
Corneal neovascularization	circZNF609	Upregulated in the corneal epithelium of corneal neovascularization models.	Promote the angiogenic function of HCEKs *in vivo*. Aggravate the corneal angiogenesis *in vivo*.	miR-184-AKT/β-catenin/VEGF	Wu et al., 2020
	circKIFAP3	Downregulated in alkali burn-induced neovascularization corneal models and patients’ vascularized corneas.	Suppress the angiogenic function of HUVECs *in vitro*.		Zhou Y.-F. et al., 2019
Pterygium	circ_0085020	Upregulated in pterygium tissues	Promote the viability, proliferation, migration, and antiapoptosis under UV exposure of pterygium fibroblasts and epithelial cells *in vitro*.		Li X.-M. et al., 2018
PVR	circ_0043144	Upregulated in serum ERMs of PVR patient.	Promote the proliferation, migration, and secretion ability of RPE cells *in vitro*.		Yao et al., 2019
Retinoblastoma	circ_0001649	Downregulated in RB cell lines and RB tissues of patients.	Suppress the proliferation and antiapoptosis of RB cells *in vitro*. Suppress the xenograft tumor growth of RB *in vivo*.	AKT/mTOR	Xing et al., 2018
	circ−0075804	Upregulated in RB cell lines and RB tissues of patients.	Promote the proliferation and antiapoptosis of RB cells *in vitro*. Promote the xenograft tumor growth of RB *in vivo*.	E2F3, HNRNPK	Zhao et al., 2020
Conjunctival melanoma	circMTUS1	Upregulated in CM tissues.	Promote the proliferation of CM cell lines *in vitro*. Promote the xenograft tumor growth of CM *in vivo*.		Shang et al., 2019

*DR, Diabetic retinopathy; FVM, fibrovascular membrane; HRVECs, human retinal vascular ECs; MMP-2, matrix metallopeptidase 2; STAT3, signal transducer and activator of transcription 3; XIAP, x-linked inhibitor Of apoptosis; HIPK3, homeodomain interacting protein kinase 3; HG, high glucose; DM, diabetes mellitus; VEGF, vascular endothelial growth factor; FZD4, frizzled-4; WNT2, Wnt family member 2; ZNF, zinc finger protein; HUVECs, human umbilical vein endothelial cells; MEF2A, myocyte-specific enhancer factor 2A; DMNT3B, DNA (cytosine-5-)-methyltransferase 3 beta; ECs, endothelial cells; HRMECs, human retinal microvascular ECs; BAMBI, BMP and activin membrane bound inhibitor; PWWP2A, PWWP domain containing 2A; SIRT1, sirtuin 1; SP1, specificity protein 1; CSPG4, chondroitin sulfate proteoglycan 4; LOXL2, lysyl oxidase homolog 2; CDK2, cyclin-dependent kinase 2; ROP, Retinopathy of prematurity; OIR, oxygen?induced retinopathy; AMD, Age-related macular degeneration; ZBTB44, zinc finger and BTB domain containing 44; VCAM1, vascular cell adhesion protein 1; nAMD, neovascular AMD; CNV, choroidal neovascularization; NR3C1, nuclear receptor subfamily 3 group C member 1; RPE, retinal pigment epithelium; PTEN, phosphatase and tensin homolog on chromosome ten; mTOR, mammalian target of rapamycin; ZRANB1, zinc finger RAN-binding domain containing 1; RGCs, retinal ganglion cells; RUNX2, runt-related transcription factor 2; METRN, meteorin; ARC, age-related cataracts; EMT, epithelial-mesenchymal transition; HLECs, human lens epithelial cells; CRYAA, crystallin alpha A; HCEKs, corneal epithelial keratinocytes; KIFAP3, kinesin-associated protein 3; UV, ultraviolet radiation; PVR, proliferative vitreoretinopathy; ERMs, epiretinal membranes; RB, retinoblastoma; E2F3, E2F transcription factor 3; HNRNPK, heterogeneous nuclear ribonucleoprotein K; MTUS1, microtubule-associated scaffold protein 1; CM, conjunctival melanoma.*

## Properties of Circular RNA

### Abundance

At first, most discoveries of circular isoforms in the genes of live beings were perceived as mis-splicing occurrences (Cocquerelle et al., 1993). A sea change occurred in 2012 when Salzman et al. (2012) applied RNA-sequencing and computation analysis to identify the global expression of circRNAs in diverse human cell lines. Since then, circRNAs have been found to be abundant and widespread not only in metazoans including mice, *Drosophila*, and zebrafish but also in protists, fungi, and plants (Li X. et al., 2018). Although circRNAs generally have a low expression level (Jeck et al., 2013; Memczak et al., 2013; Salzman et al., 2013; Guo et al., 2014), in some cases, their abundance can exceed that of cognate linear mRNAs due to higher expression or spatiotemporal accumulation (Ashwal-Fluss et al., 2014; Westholm et al., 2014; Conn et al., 2015; Rybak-Wolf et al., 2015; Szabo et al., 2015; Venø et al., 2015; You et al., 2015).

### Specificity

The expression patterns of circRNAs are very diverse in different cell and tissue types, as well as developmental stages (Han et al., 2018). As well known, the SRY gene in adult mouse testes produces a circular isoform exclusively instead of linear mRNA (Capel et al., 1993), and the expression of circRNAs is generally induced in the embryonic development stage (Westholm et al., 2014; Szabo et al., 2015). In humans, pigs, and mice, circRNAs are much more abundant in the brains, where the circRNA modulation is correlated with neuronal development and differentiation, homeostatic neuronal activity, and synaptogenesis (Rybak-Wolf et al., 2015; Szabo et al., 2015; Venø et al., 2015; You et al., 2015). Furthermore, the transcription of circRNAs is also dynamic during different biological processes, such as epithelial-mesenchymal transition (EMT) (Conn et al., 2015).

### Conservation

Wang et al. (2014) argued that the gene expression of circRNAs was either highly conserved or the result of repeated convergent evolution. Orthologous genes in mice and humans were estimated to produce approximately 5–30% of conserved circRNAs (Jeck et al., 2013; Memczak et al., 2013; Guo et al., 2014; Rybak-Wolf et al., 2015). In addition, nearly 20% of porcine splice sites (ss) correlated with circRNA production were functionally conserved between mice and humans (Venø et al., 2015). Moreover, mechanisms of circRNA biogenesis and similar structural features of circRNA were also judged to be conserved (Jeck et al., 2013; Ashwal-Fluss et al., 2014; Liang and Wilusz, 2014; Zhang et al., 2014). It is worth noting that circular exons were better conserved than flanking exons, which were more likely to be flanked by introns with reverse complementary matches (RCMs) (Rybak-Wolf et al., 2015).

### Stability

Ribonuclease R (RNase R) is a 3′ to 5′ exoribonuclease and is capable of catalyzing the degradation of linear RNA, so the absence of 3′ and 5′ terminals in circRNAs allows them to resist RNase R degradation (Suzuki, 2006). Jeck et al. (2013) found circRNAs had half-lives exceeding 48 h, while linear transcripts occurred for less than 20 h. Thus, circRNAs are much more stable in cells, tissues, blood, saliva, urine, exosomes, etc., than their linear counterparts due to their covalently closed loop structure. In the aging process of the brain, stable circRNAs can accumulate and exist in richer abundance in quiescent and postmitotic cells (Westholm et al., 2014; Rybak-Wolf et al., 2015).

These characteristics make circRNA production another special feature of genomes rather than transcriptional noise. Besides, circRNAs are delicately regulated, play a vital role in living organisms, and serve as potential biomarkers in bodily fluid for disease diagnosis.

## Biogenesis of the Circular RNA

### Classification

According to recent studies, most of the circRNAs in eukaryotes can be divided into three subtypes depending on their components (Figure 1A; Lasda and Parker, 2014; Liu J. et al., 2017): (1) exonic circRNAs (ecircRNAs): generated by one or more exons circularization; (2) exon-intron circRNAs (EIciRNAs): circularized from exons with introns reserved; (3) intronic circRNAs (ciRNAs): composed only of introns. Different from the canonical splicing, circRNAs are generally formed by the joining of an upstream 3′ acceptor and a downstream 5′ donor, which is called “back-splicing” (Lasda and Parker, 2014). The sequence of ciRNAs is assembled head-to-tail by a 2′, 5′ phosphodiester bond (Zhang et al., 2013).

Furthermore, there are five subsets of circRNA classification based on the location relationship between circRNAs and adjacent coding RNA (Liu J. et al., 2017): (1) and (2) “exonic” and “intronic”: formed by exons and introns, respectively; (3) “antisense”: transcribed into the opposite strand from their gene locus being overlapped by linear isoforms; (4) “sense overlapping”: produced by the same gene locus but does not belong to the “exonic” or “intronic” circRNA; (5) “intergenic”: transcribed from the location that is outside the gene loci.

### Mechanism and Regulation

The process of back-splicing requires canonical splicing signals as well as spliceosome machinery (Ashwal-Fluss et al., 2014; Starke et al., 2015; Liang et al., 2017). Restricting canonical pre-mRNA splicing processes by inhibiting or deleting core spliceosome complexes has been reported to shift the steady-state production of linear mRNAs toward the preferred output of circRNAs (Liang et al., 2017; Wang et al., 2019). Thus, canonical splicing is a default choice for gene splicing in most cases.

Two classical models explain the biogenesis of circRNAs according to the order of canonical and back-splicing (Figures 1B,C):

(1) The “direct back-splicing” model or RNA pairing model (Chen and Yang, 2015; Li X. et al., 2018; Han et al., 2018): Back-splicing occurs first, then a circular structure with intermediate exons and introns are generated directly and are processed into the final product. In this model, RNA pairing across flanking introns brings back-splicing sites into closer proximity and promotes efficient biogenesis of circRNAs. RNA pairing can be driven by repetitive flanking intron sequences, such as inverted repetitive Alu elements, or RCMs (Liang and Wilusz, 2014; Zhang et al., 2014; Ivanov et al., 2015; Kramer et al., 2015). In addition, some double-stranded RBPs, such as immune factors NF90/110, can facilitate RNA pairing formation and some RBPs without dsRNA binding domains, such as muscleblind (MBL) and quaking (QKI), can bind to specific targets in introns (Ashwal-Fluss et al., 2014; Conn et al., 2015; Li et al., 2017; Liu J. et al., 2017). The QKI dimerization at binding sites in the flanking introns can increase the production of many circRNAs (Conn et al., 2015). However, adenosine deaminase acting on RNA 1 (ADAR1) can recognize and diminish the double-stranded RNA (dsRNA) pairing structure through A-to-I editing in dsRNA regions, exerting opposite influence on the biogenesis of circRNAs (Ivanov et al., 2015; Rybak-Wolf et al., 2015).

(2) The “exon skipping” or “lariat-driven” model (Barrett et al., 2015; Chen and Yang, 2015; Li X. et al., 2018; Han et al., 2018): In this model, canonical splicing occurs first, then a lariat structure is generated with skipping exons and introns. Following introns being dislodged, retained components are ligated head-to-tail to produce ecircRNAs or EIciRNAs by internal splicing. Note that a lariat intron, which has a consistent motif containing a seven-nucleotide (nt) Adjacent bases of guanine and uracil (GU)-rich element at the 5′ ss and an 11-nt C-rich element at the 3′ branchpoint site, can be generated and can avoid debranching and degradation (Zhang et al., 2013). Then stable ciRNAs are produced from the lariat intron after removing the 3′ tail in the downstream of the branchpoint site (Zhang et al., 2013). Interestingly, a circRNA from the *Arabidopsis* SEPALLATA3 gene can bind to the cognate DNA locus and form a DNA:RNA hybrid (R-loop), promoting further exon skipping (Conn et al., 2017).

In a single gene locus, alternative splicing occurs in both models to produce various circRNAs containing different combinations of introns and exons. And some circRNAs can be generated from readthrough transcription events (Liang et al., 2017).

### Subcellular Localization

Once generated, the vast majority of circRNAs are located in the cytoplasmic compartment (Salzman et al., 2012, 2013; Jeck et al., 2013; Memczak et al., 2013), but some subsets, such as EIciRNAS and ciRNAs, remain in the nucleus, which may regulate gene transcription or expression (Zhang et al., 2013; Li Z. et al., 2015; Venø et al., 2015; Conn et al., 2017). The translocation of circRNAs from the nucleus to cytoplasm through the nuclear pore complex relies on proteins – Hel25E homologs (UAP56/URH49 in humans) and is based on a length-dependent mechanism (Figure 1D; Huang et al., 2018). In humans, URH49 and UAP56 modulate the localization of short (<400 nt) and long (>1,200 nt) circRNAs, respectively (Huang et al., 2018). However, the localization of circRNAs is not stationary. You et al. (2015) showed circZEB1 was transited from the cytoplasm and perinuclear sites at E60 to the nucleus at E80, and many circRNAs were enriched in synapses. Moreover, circRNAs can be exported through exosomes and execute their functions (Figure 1E). And more than 1,000 circRNAs have been found enriched in human serum exosomes (Li Y. et al., 2015).

Adjustable expression levels and subcellular localization of circRNAs are indicated to be tightly correlated with their function manipulation. While mechanisms that modulate the biogenesis and translocation of many circRNAs have been discovered, molecular details are still poorly understood.

## Functions of Circular RNA

### MicroRNA Sponges or Reservoirs

The miRNA, a type of short single-stranded ncRNA, is capable of binding to the 3′ untranslated regions (UTRs) of protein-coding mRNAs and inhibiting their translation by forming an RNA-induced silencing complex (RISC) with the argonaute 2 (Ago2) (Baek et al., 2008). CircRNAs contain miRNA recognition elements (MREs) and rescue mRNAs from miRNA combination through competitive binding. CDR1as/ciRS-7, containing more than 70 MREs of miR-7, was the first circRNA to be identified as a miRNA sponge (Figure 1.1; Hansen et al., 2013a; Memczak et al., 2013). However, the cleavage of ciRS-7, triggered by miR-671 in an Ago2-slicer-dependent manner (Hansen et al., 2011), could lead to immediate spatiotemporal activation of miR-7 and repression of mRNA targets (Hansen et al., 2013b). Thus, ciRS-7 may function as the reservoir or storage of miR-7 and transport it to the specific subcellular location (Hansen et al., 2013b; Memczak et al., 2013).

### Interaction With Proteins

The interaction between proteins and circRNAs is much intricate, and circRNAs can serve as transporters, decoys, or scaffolds for proteins (Figure 1.2; Patop et al., 2019). CircFoxo3, for example, can have a high binding affinity to some transcription factors and inhibit their nuclear translocation and anti-tress function during cardiac stress (Du et al., 2017b). Another study found circFoxo3 facilitated the inhibition of CDK2 by p21 by generating a ternary complex with p21 and CDK2 (Du et al., 2016). It is first to show that circRNAs can act as a scaffold to form the protein-protein complex and modulate protein interactions (Du et al., 2016). And it has been determined that dynamic tertiary structures of circRNAs enable them to interact with various proteins (Du et al., 2017a, c). Many circRNAs are likely to create a molecular reservoir for antiviral proteins NF90/110 before a viral infection occurs. This behavior supports the hypothesis that circRNAs may regulate activities of proteins *via* cooperative actions (Li et al., 2017).

### Regulation of Transcription, Splicing, and Translation

Some circRNAs, such as EIciRNAs and ciRNAs, are positioned in the nucleus and regulate gene transcription and splicing (Figures 1.3a,b). For example, based on a specific RNA-RNA interaction, EIciRNAs-U1 snRNP complexes combine with RNA Pol II to promote cognate gene transcription (Li Z. et al., 2015). In addition, ciRNAs can perform cis-regulation by interacting with Pol II, and the depletion of ciRNAs can lead to a significant downregulation of parent gene transcription (Zhang et al., 2013). CircMBL, on the other hand, can strongly bind to MBL and promote back-splicing to produce more circMBL instead of MBL mRNA, which forms a negative-feedback regulation loop (Ashwal-Fluss et al., 2014). CircRNAs can also regulate mRNA translation (Figure 1.3c). For instance, circPABPN1 can reduce the expression of PABPN1 by competing with its cognate linear mRNA for human antigen R (HuR, an RBP) (Abdelmohsen et al., 2017).

### Translation Into Peptides or Proteins

Synthetic circRNAs implanted with an internal ribosome entry site (IRES) have already been proven to be translated *in vivo* and *in vitro* (Chen and Sarnow, 1995; Wang and Wang, 2015). CircZNF609 was the first natural circRNA discovered to translate to protein through the splicing-dependent and cap-independent mechanisms (Legnini et al., 2017). It was also discovered that circMBL from fly heads encodes a protein (Pamudurti et al., 2017). Both translatable circRNAs have IRES embedded within their sequences. The translation of circRNAs also can be driven by N^6^-methyladenosine (m^6^A) modification in a cap-independent manner (Figure 1.4), which requires initiation factor eIF4G2 and m^6^A reader YTHDF3 (Yang et al., 2017).

### Generation of CircRNA-Derived Pseudogenes

Pseudogenes are important regulators at the DNA, RNA, or protein level in diverse physiological and pathological processes (Xiao-Jie et al., 2015). Similar to mRNAs, circRNAs have the potential to be reverse transcribed and integrated into their host genomes to generate pseudogenes (Figure 1.5). Dong et al. (2016) determined that circRNA-derived pseudogenes may reshape genome architecture by providing additional CCCTC binding factor-binding sites. However, the mysterious generation mechanism and functions of circRNA-derived pseudogenes are still unknown.

### Regulate Epigenetic Alterations

A few circRNAs have been found to affect epigenetic modifications by acting on DNA or histone methylation (Figure 1.6; Chen et al., 2018; Su et al., 2019). For instance, circFECR1 can bind to the promoter of FLI1 in cis and lead to DNA hypomethylation by recruiting a demethylase TET1 (Chen et al., 2018). It can also bind to and downregulate trans DNMT1, a methyltransferase that is essential for maintaining DNA methylation (Chen et al., 2018). Through epigenetic modification, circFECR1 positively activates the FLI1 and underlines the deep mechanism of tumor growth and metastasis (Chen et al., 2018, 1). Several circRNAs can indirectly regulate histone methylation by sponging miRNAs that inhibit the expression of an N-methyltransferase enzyme called EZH2 (Su et al., 2019).

Although most of the identified functional circRNAs act as miRNA sponges, they have varied biological functions that support our recognition of the importance of circRNAs in parental cell activities. Additionally, exosomal circRNAs may regulate biological processes in distant cells and tissues (Li Y. et al., 2015; Shi et al., 2020).

## Involvement of Circular RNA Research in Ocular Diseases

Recently, in a study conducted by Sun et al. (2019), retinal circRNA repertoires showed conservation and variation across six vertebrate species, and the expression pattern of circRNAs had specific signatures in different stages of retinal development and degeneration (Chen X.-J. et al., 2020). The deficiency of circTulp4 in mice caused the mouse retinas to have abnormal structures and defective functions (Chen X.-J. et al., 2020). These studies further convince us to highlight the potential key biological functions of circRNAs in regulating eye development as well as ocular diseases. In this part, we focus on accumulative evidence that has identified that circRNAs play a crucial role in the pathogenesis and progression of some ocular diseases (Table 1). CircRNAs can serve as potentially diagnostic, progressive, and prognostic biomarkers for these diseases, and it is promising to treat them by intervening circRNA expression.

### Retinal Diseases

#### Diabetic Retinopathy

Diabetic retinopathy (DR) is a common microvascular complication among people with diabetes mellitus (DM) and is one of the main causes of visual damage and blindness for them (Yau et al., 2012). In DM, diabetes-related stresses can cause retinal vasculatures to undergo early and prevalent damage, and disequilibrium of endothelial cells (ECs) can increase vascular exudation and macula edema and promote retinal angiogenesis (Duh et al., 2017). The combination of abnormal vascular endothelial growth factor (VEGF) expression and other factors, such as advanced glycation end products, oxidative stress, activated protein kinase C, and Phosphatidylinositol 3-kinase (PI3K)/Akt signaling pathways, has also been identified to underlie vascular dysfunction in DR (Schalkwijk and Stehouwer, 2005; Potenza et al., 2009).

**FIGURE 2:**
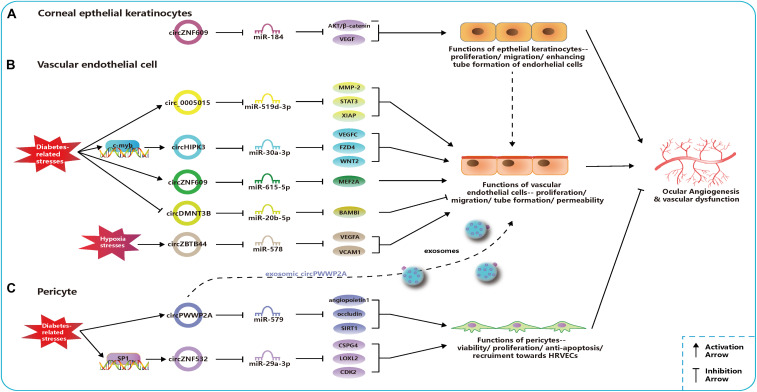
Related circular RNAs (circRNAs) in the pathogenesis of ocular neovascular diseases. **(A)** In corneal epithelial keratinocytes, circZNF609 regulates the function of epithelial cells and promote the ocular angiogenesis through miR-184-AKT/β-catenin/vascular endothelial growth factor (VEGF) network. **(B)** In vascular endothelial cells, networks of circ_0005015-miR-519d-3p-matrix metallopeptidase (MMP)-2/STAT3/X-linked inhibitor of apoptosis protein (XIAP), circHIPK3-miR-30a-3p-VEGFC/FZD4/WNT2, circZNF609-miR-615-5p-MEF2A, circDMNT3B-miR-20b-5p-BMP, and activin membrane bound inhibitor (BAMBI), and circZBTB44-miR-578-VEGFA/vascular cell adhesion molecule (VCAM)1 regulate the function of endothelial cells and the ocular angiogenesis. Circ_0005015, circHIPK3, and circZNF609 can promote angiogenesis and be upregulated under diabetes-related stresses, which is opposite to circDMNT3B. CircZBTB44 can promote angiogenesis and be upregulated under diabetes-related stresses. **(C)** In pericytes, networks of circPWWP2A-miR-579-angiopoietin1/occludin/sirtuin (SIRT)1 and circZNF532-miR-29a-3p-CSPG4/LOXL2/CDK2 regulate the function of pericytes and the ocular angiogenesis. CircPWWP2A and circZNF532 can inhibit the angiogenesis and maintain the normal vascular structure. CircPWWP2A is also packaged into exosomes and transported from pericytes to the endothelial cells.

Circ_0005015 has been identified as a putative circRNA biomarker in DR, which is significantly increased in the plasma, vitreous samples, and preretinal fibrovascular membranes (FVMs) of people with DR (Zhang S.-J. et al., 2017). In addition, circ_0005015 can promote the retinal endothelial angiogenesis by working as the miR-519d-3p sponge, which regulates the expression of matrix metallopeptidase (MMP)-2, signal transducer and activator of transcription (STAT)3, and X-linked inhibitor of apoptosis protein (XIAP) (Figure 2; Zhang S.-J. et al., 2017). Similar to circ_005015, overexpressed circHIPK3 promotes cell viability, proliferation, migration, and tube formation in human retinal vascular ECs (HRVECs) and aggravates diabetic retinal vascular dysfunction by increasing vascular leakage and the number of the acellular capillary (Shan et al., 2017). The transcription factor c-myb is responsible for the upregulation of circHIPK3 under diabetes-related stresses, and then the regulatory network of circHIPK3-miR-30a-3p-VEGFC/FZD4/WNT2 is activated (Figure 2; Shan et al., 2017). cZNF609 is another highly expressed circRNA in patients with DR, and its defect suppresses pathological angiogenesis in the DR model (Figure 2; Liu C. et al., 2017). Conversely, circDNMT3B has a decreased concentration in FVMs and human retinal microvascular ECs (HRMECs) under the high-glucose (HG) treatment. MiR-20b-5p, targeted by circDNMT3B, has been found to positively regulate endothelial angiogenesis through modulating the expression of BAMBI (Figure 2; Zhu et al., 2019). Earlier works have shown that BAMBI is involved in capillary growth regulation and angiogenesis (Guillot et al., 2012).

The dysfunction of pericytes is a leading pathologic feature of inchoate DR and the unbalanced pericyte-EC crosstalk induces the dysfunction of the retinal microvasculature system in DR (Hammes et al., 2002; Armulik et al., 2005). Researchers have verified cPWWP2A and cZNF532 are attractive targets for alternative therapies of pericyte degeneration and DR (Liu et al., 2019; Jiang et al., 2020). Upregulated expressions of cPWWP2A and cZNF532 were found in pericytes under DM-related stresses and in clinical samples from people with DR, but not in ECs, which were verified to antagonize the diabetes-induced disruption to vascular homeostasis (Liu et al., 2019; Jiang et al., 2020). And the severity of DR was also correlated to rising levels of cZNF532 in the vitreous (Jiang et al., 2020). The SP1, a transcription factor activated in diabetic condition, could bind to the promoter of cZNF532 and take charge of increasing cZNF532 expression (Jiang et al., 2020). In pericytes, cZNF532 can promote the expression of CSPG4, LOXL2, and CDK2 by sequestering the miR-29a-3p binding sites, which results in increased cell viability, proliferation, and recruitment toward HRVECs, ameliorated diabetic stress-induced cell apoptosis, and decreased the macromolecular permeability (Figure 2; Jiang et al., 2020). However, manipulating the cZNF532 expression had almost no effect on HRVECs (Jiang et al., 2020). cPWWP2A had similar effects to those of cZNF532 on the regulation of pericyte functions *via* a different network—cPWWP2A-miR-579-angiopoietin1/occludin/SIRT1, but, unlike cZNF532, it was able to indirectly modulate HRVEC angiogenic activities and pericyte-EC crosstalk through a paracrine approach in exosomes from pericytes (Figure 2; Liu et al., 2019). In conclusion, the overexpression of cZNF532 and cPWWP2A or the silencing of miR-29a-3p and miR-579 was found to alleviate the diabetes-related damage to pericyte functions *in vitro* and retinal vasculatures *in vivo*, as it resulted in an increase in pericyte coverage and a reduction of vascular leakage, the acellular vascular area, and the amount of microaneurysm (Liu et al., 2019; Jiang et al., 2020).

Another two studies identified altered expression profiles of circRNAs in serum samples and the vitreous humor of people with DR, which could serve as a foundation for future studies on the role of circRNAs in DR (Gu et al., 2017a; He et al., 2020).

#### Retinopathy of Prematurity

Retinopathy of prematurity (ROP), a retinal vasoproliferative disease, primarily occurs in premature infants and may cause blindness (Chen and Smith, 2007; Chan-Ling et al., 2018). The pathogenesis of ROP is known to have two phases: Phase 1 is hyperoxia-induced incomplete retinal vessel growth after premature birth, and Phase 2 is hypoxia-driven pathological vessel proliferation (Chen and Smith, 2007; Chan-Ling et al., 2018). Current ablation treatments, the injection of anti-VEGF agents, and other relatively effective treatments contribute to reduced blindness and improved long-term prognosis for people with ROP (Chan-Ling et al., 2018).

The oxygen-induced retinopathy (OIR) newborn mouse model is widely used for mimicking the development of ROP and studying the pathogenesis of retinal neovascularization (Scott and Fruttiger, 2010). Using the OIR model, it was found that cZNF609 was downregulated at the vaso-obliteration stage (Postnatal Days 7–12), but it was upregulated at the neovascularization stage (P12–17) (Liu C. et al., 2017). The reduction in cZNF609 dramatically shrunk the avascular area and suppressed pathologic angiogenesis *in vivo* at P12 and P17 (Liu C. et al., 2017). Decreased cZNF609 also positively regulated human umbilical vein endothelial cell (HUVEC) functions by targeting miR-615-5p-MEF2A network (Figure 2). Therefore, MEF2A silencing can have a mimicking effect on HUVECs as cZNF609 (Liu C. et al., 2017). Two more pieces of research contain a microarray analysis of circRNAs based on the OIR model, providing further research targets for ROP pathogenesis (Cao et al., 2019; Zhou H. et al., 2019).

#### Age-Related Macular Degeneration

##### Exudative age-related macular degeneration

Age-related macular degeneration (AMD) is one of the leading causes of irreversible blindness in the elderly. AMD is classified into two subtypes–exudative AMD and atrophic AMD–according to whether choroidal vessels disruptively invade the retina (Lim et al., 2012). Exudative AMD, also called neovascular AMD (nAMD) or wet AMD, is characterized by choroidal neovascularization (CNV) leading to retinal pigment epithelium (RPE) rupture, leaking lipids and blood, and fibrous scarring (Lim et al., 2012). Intraocular injection of anti-VEGF agents is the current main treatment for nAMD (Bloch et al., 2012). Whether dysregulated circRNA contributes to developing nAMD is being studied recently.

Liu et al. (2020) investigated circRNA expression profiles using the laser-induced CNV mouse model and constructed circRNA-miRNA-mRNA networks potentially regulating the CNV development. Zhou et al. (2020) identified that cZBTB44 could regulate the pathogenesis of CNV. They also found that cZBTB44, as well as VEGFA and vascular cell adhesion molecule (VCAM)1, was significantly upregulated not only in chorioretinal vascular ECs under hypoxic conditions and CNV lesions from mouse models but also in the aqueous humor of patients with nAMD (Zhou et al., 2020). Moreover, the silencing of cZBTB44 led to the reduction of the CNV lesion area *in vivo* and reduced the size of the choroidal capillary area and the sprouting area in choroidal sprouting assay *ex vivo* (Zhou et al., 2020). cZBTB44 can promote endothelial angiogenic effect by sponging the miR-578 and then modulating the level of VEGFA/VCAM1 indirectly in choroid-retinal ECs (Figure 2; Zhou et al., 2020).

##### Atrophic age-related macular degeneration

Atrophic AMD, also called dry AMD, is characterized by persistent atrophy of the RPE, choriocapillaris, and photoreceptors (Lim et al., 2012). RPE cells can maintain retinal functions through vital activities, such as light absorption, nutrient or metabolic waste transportation, and growth factor secretion (Strauss, 2005; Chen X. et al., 2020). RPE abnormalities, like dedifferentiation and degeneration, underlines the pathogenesis of early atrophic AMD, and the strategies for RPE protection will be a promising therapy in the future (Strauss, 2005; Lim et al., 2012).

A recent study revealed that the expression of circNR3C1 was downregulated in serum samples of people with atrophic AMD patients and RPE cells under oxidative stress (Chen X. et al., 2020). CircNR3C1 was found to protect RPE functions *via* regulating the miR-382-5p-phosphatase and tension homolog on chromosome ten (PTEN)-AKT/mammalian target of rapamycin (mTOR) networks (Chen X. et al., 2020). More specifically, insufficient endogenous circNR3C1 expression could disturb RPE ultrastructure, reduce RPE markers, interrupt phagocytosis, and promote RPE proliferation (Chen X. et al., 2020). PTEN, an inhibitor of the AKT/mTOR signaling pathway, enables RPE cells to function normally, but the activation of the AKT/mTOR pathway triggers RPE dedifferentiation and retinal degeneration (Kim et al., 2008; Lee et al., 2011; Zhao et al., 2011; Jiang et al., 2016).

#### Glaucoma

As circRNAs are abundant, well conserved, and dynamically expressed in the central nervous system (Rybak-Wolf et al., 2015; You et al., 2015), they play a vital role in the modulation of neurodegeneration disorders (Kumar et al., 2017). Glaucoma is an irreversible and progressive retinal neurodegenerative disease and is the second leading cause of blindness worldwide (Kingman, 2004). It is caused by the progressive loss of retinal ganglion cells (RGCs) (Weinreb et al., 2014). The only method to delay or halt the progression of glaucoma and help RGC survival is to reduce intraocular pressure through surgery and medication (Weinreb et al., 2014).

Although the pathogenesis of glaucoma is highly complex and still unclear, two recent studies have revealed that circRNAs played an important role in glaucoma (Wang et al., 2018a, b). Wang et al. determined that the expression levels of Wang et al. (2018a) and Wang et al. (2018b) were upregulated by elevating the intraocular pressure (IOP) in the rat microbead injection-induced glaucoma model, which was consistent with the detection results in aqueous humor from people with glaucoma. *In vivo*, knockdown of cZNF609 and cZRANB1 inhibited the reaction of retinal gliosis and facilitated the survival of RGCs, but it did not affect the amacrine cells, photoreceptors, or bipolar cells. *In vitro*, silencing cZNF609 or cZRANB1 suppressed the viability, proliferation, and activation of Müller cells. However, the silencing indirectly regulated RGC function by reducing the proapoptotic effects of Müller cells on RGCs under oxidative stress and glutamate toxicity stress. cZNF609 was validated to function as the sponge for miR-615, targeting METRN. In addition, the cZRANB1-miR-217-RUNX2 network was uncovered in Müller cells. Moreover, circRNAs showed abnormal expression patterns in a much earlier stage, before the onset of retinal degeneration diseases, in *in vivo* models (Chen X.-J. et al., 2020). The RUNX2 is involved in mammalian neural development, and METRN influences glial cell differentiation and axonal network formation (Nishino et al., 2004; Zagami et al., 2009). A therapy targeting cZNF609 and cZRANB1 that would indirectly regulate these target genes may be promising as a glaucoma treatment.

### Cataract

#### Age-Related Cataract

Cataract is characterized by the loss of transparency of the human lens. Severe opacification leads to visual impairment or blindness at last. Cataracts can be categorized based on the etiology: age-related cataracts (ARCs), congenital cataracts, and secondary cataracts (cataracts caused by other factors, such as diabetic cataracts) (Liu Y.-C. et al., 2017). The etiopathogenesis of ARC can include the accumulation of insoluble crystallin and dysregulated biological activities of human lens epithelial cells (HLECs), such as abnormal cell growth, cell death, and differentiation (Michael and Bron, 2011). Although cataract surgery is an efficient way to manage cataracts, the rapidly increasing demand for cataract surgery poses a great economic burden on society (Rao et al., 2011; Liu Y.-C. et al., 2017). A more precise molecule mechanism is being studied to find promising alternative pharmacological means for ARC treatment.

Liu et al. (2018) found a general decrease in the expression of circHIPK3 in all three subtypes of ARC (cortical, nuclear, and posterior subcapsular ARC). CircHIPK3 is an abundant and highly conserved circRNA that is involved in a series of physiological or pathological activities (Xie et al., 2020). Silencing circHIPK3 led to the suppression of various cell activities in primary cultured HLECs, such as cell viability, proliferation, EMT, and anti-apoptosis under oxidative stress (Liu et al., 2018). The function of circHIPK3 was manipulated through the circHIPK3-miR-193a-3p-CRYAA axis in HLECs. The CRYAA encodes the αA-crystallin, which is necessary for the maintenance of lens transparency and lens epithelium survival (Andley, 2009).

#### Diabetic Cataract

Cataract often occurs at an earlier age and keeps progressing faster in people with DM (Harding et al., 1993). The proliferation, apoptosis, and autophagy of HLECs are often induced by the hyperglycemic and hyperosmotic microenvironment combined with impaired antioxidant systems (Pollreisz and Schmidt-Erfurth, 2010; Zhang L. et al., 2017). Cataract surgery for patients with DM should be carried out more cautiously and has higher complication rates (Pollreisz and Schmidt-Erfurth, 2010). Interventions that target the pathogenic mechanism of diabetic cataract to delay or confine its onset and progression remain not well understand.

Fan et al. (2019) identified the expression profiling of circRNAs in people with DR first. They detected that circKMT2E was significantly upregulated in circRNAs of the anterior capsular tissues of the lens in those patients, which was the opposite of the results for the promising miRNA target—miR-204-5p, which is related to cell autophagy (Cost and Czyzyk-Krzeska, 2015; Zhang L. et al., 2017; Fan et al., 2019). The autophagy-related circKMT2E/miRNA/mRNA interaction network was only putatively depicted in this study, which may affect the pathogenesis of diabetic cataract (Fan et al., 2019). Thus, the function of circKMT2E in cataract is still imprecise.

### Corneal Diseases

The cornea is a transparent and avascular part of the eye when it is healthy, while the corneal limbus has many blood vessels to support the survival of stem cells (Chang et al., 2001). However, in people with immunologic, traumatic, or infectious disorders on the ocular surface, corneal neovascularization results from newborn blood vessels from the limbus invading the cornea (Casey and Li, 1997; Chang et al., 2001). Corneal neovascularization often leads to profound vision loss and increases the risk of graft failure and rejection after corneal transplantation (Chang et al., 2001; Bachmann et al., 2010).

The expressions of cZFP609 (cZNF609 in humans) and cKifap3 are the most upregulated and downregulated circRNAs, respectively, in the alkali burn-induced corneal neovascularization model, with results consistent with those of patients’ vascularized corneas (Zhou Y.-F. et al., 2019). The phenomenon of persistent upregulated cZNF609 and downregulated miR-184 was also discovered in the rat cornea after corneal suture surgery (Wu et al., 2020). *In vitro*, cKifap3 knockdown improved the angiogenic function of HUVECs (Zhou Y.-F. et al., 2019). In addition, cZNF609 was found to promote angiogenesis by acting as a sponge for miR-184 and activating downstream AKT/β-catenin/VEGF in human corneal epithelial keratinocytes (HCEKs) (Figure 2). This circRNA was revealed to increase the proliferation and migration of HCEKs and enhance the tube formation of ECs (Wu et al., 2020). Thus, topical administration to upregulate the miR-184 or attenuate the cZNF609 was effective in decreasing corneal neovascularization in corneal sutured rats (Wu et al., 2020).

As mentioned above, enhanced expressions of circ_0005015, cZNF609, circHIPK3, and cZBTB44 or decreased expressions of circDMNT3B can promote the angiogenic function of vascular endothelial cells. The overexpression of cZNF609 can strengthen the angiogenesis effect in corneal epithelial keratinocytes, and the overexpression of cPWWP2A or cZNF532 can regulate pericyte functions and alleviate retinal vascular dysfunction *in vivo*. These dysregulated circRNAs, together with their regulatory networks, disrupt the balance of angiogenic and angiostatic factors and modulate the cell activities of endothelial cells, epithelial keratinocytes, and pericytes (Figure 2). These findings provide new insights into the pathogenesis of vascular eye diseases.

### Ocular Surface Diseases

Pterygium, a common ocular surface disorder, is characterized by benign noncancerous overgrowth of conjunctiva over the sclera (Liu et al., 2013). Visual function is significantly affected if the hyperplastic fibrovascular tissue invades the cornea or causes inflammation (Liu et al., 2013). Genetic factors (e.g., DNA repair, cell proliferation and migration, and angiogenesis) and environmental factors [e.g., human papillomavirus infection and chronic ultraviolet radiation (UV) exposure] underlie the pathogenesis of pterygium (Liu et al., 2013). However, the precise molecular mechanism of pterygium formation is not clear, and pterygium has a relatively high recurrence ratio with the current treatments (Hacıoğlu and Erdöl, 2017).

In a recent study, 669 circRNAs were found to be abnormally expressed in pterygium tissues (Li X.-M. et al., 2018). Further analysis revealed that the most enriched biological process and regulatory network of dysregulated circRNAs are the extracellular matrix organization and focal adhesion signaling pathways, respectively, which are highly correlated with the pathogenesis of pterygium (Li X.-M. et al., 2018). The researchers focused on significantly upregulated circ_0085020 (circLAPTM4B) and found that its silencing weakened cellular viability, proliferation, and migration of pterygium fibroblasts and pterygium epithelial cells but increased UV-induced apoptosis (Li X.-M. et al., 2018). More researches are required to more thoroughly understand the regulatory network of circ_0085020.

### Vitreous Diseases

Proliferative vitreoretinopathy (PVR) commonly occurs after retinal detachment (RD) surgery or other intraocular surgeries due to the genesis of membranes on the surface between the detached retina and the posterior hyaloid (Pastor et al., 2016). The migration and proliferation of RPE and glial cells have been considered essential elements of the pathogenesis of PVR (Pastor et al., 2016). Proliferating membranes may lead to further tractional RD, and current surgical treatments carry the risk of retina re-detachment (Charteris et al., 2002), so it is important to prevent the development of PVR.

Yao et al. (2019) screened 91 circRNAs that were dysregulated in the epiretinal membranes (ERMs) of people with rhegmatogenous RD who were diagnosed with PVR. Among the 91 circRNAs was circ_0043144, which has a dynamic expression consistent with established PVR markers, and its circulating molecular concentration in serum samples positively increased in a PVR severity assessment, but there was no difference after the surgery for PVR (Yao et al., 2019). In *in vitro* studies, the silence of circ_0043144 induced the dysfunction of RPE cells, such as suppressed proliferation and migration and attenuated secretion of cytokines and growth factors that may prevent the formation of ERMs (Yao et al., 2019). Circ_0043144 may be able to perform as an indicator for the diagnosis and aggravation of PVR, as well as a potential therapeutic target.

### Hyperhomocysteinemia-Induced Ocular Diseases

Hyperhomocysteinemia (HHcy) is a type of metabolic disease in which there is an abnormally high level of homocysteine (Hcy) in the patient’s plasma caused by a deficiency of cystathionine-β-synthase (CBS), methionine synthase, and other factors involved in the metabolism, such as vitamins B6 and B12 (Ajith and Ranimenon, 2015). Many pieces of evidence have supported that HHcy can induce various ocular pathological changes correlated with glaucoma, cataract, retinopathy, optic neuropathy, retinal vascular diseases, and many other eye disorders (Ajith and Ranimenon, 2015).

A profile of differentially expressed circRNAs was identified in the eyes of the CBS-deficient murine model with HHcy (Singh et al., 2018). On the other hand, Singh et al. (2019) found 54 dysregulated circRNAs in the RPE cells exposed to high levels of Hcy. In the future, more functional circRNAs should be verified to help us better understand the role of circRNAs in HHcy-induced ocular diseases.

### Ocular Malignancies

#### Retinoblastoma

Retinoblastoma (RB) is a progressive intraocular cancer that occurs most commonly in young children (Dimaras et al., 2012). This has an approximate mortality ratio of 70% in pediatric patients with RB in underdeveloped countries (Dimaras et al., 2012). The genetic etiology or pathogenesis of RB is suspected to be what is known as the Knudson’s “two-hit” hypothesis. According to this hypothesis, there is first a mutation of the RB1 gene and then the second hit occurs in the RB1 alleles (Knudson, 1971). However, more research has revealed a more complex landscape of genetics and epigenetics in which other biological molecules and events affect the origin and prognosis of RB, including DNA methylation, miRNA, and circRNAs (Reis et al., 2012).

Has_circ_0001649, transcribed from an antioncogene SHPRH, is a novel cancer-associated circRNA found in several cancers, such as cholangiocarcinoma (Xing et al., 2018; Xu et al., 2018). The downregulation of circ_0001649 was found in RB tissues and human RB cell lines, and it was correlated with a larger bulk of tumors, more severe retinoblastoma, and reduced 5-year survival rate after surgeries (Xing et al., 2018). Furthermore, overexpressed circ_0001649 was shown to inhibit the proliferative ability of RB cells by modulating the signaling pathway of AKT/mTOR and slow down the xenograft growth of RB in the mouse model (Xing et al., 2018). In another study, circ_0075804 and its homologous mRNA derived from gene E2F3 were highly expressed in RB cells (Zhao et al., 2020). Interestingly, circ_0075804 was found to enhance the stability of E2F3 mRNA with the help of HNRNPK (a type of RBP) (Zhao et al., 2020). Thus, the level of E2F3 mRNA and coding protein was upregulated by circ_0075804, but E2F3 had no impact on circ_0075804 (Zhao et al., 2020). The silencing of circ_0075804 can lead to downregulated E2F3 and then inhibit its proliferation, promote apoptosis of RB cells, and slow down RB progression (Zhao et al., 2020).

Lyu et al. (2019) found that there was a correlation between the parental genes of dysregulated circRNAs in RB samples and the function of chromatin modification, which is an important part of the pathogenesis of RB. A potential regulatory axis of has_circ_0093996-miR-183-PDCD4 is predicted to play a role in RB pathogenesis (Lyu et al., 2019).

#### Ocular Melanoma

Malignant melanoma, a type of highly invasive tumor made up of melanocytes, often occurs in sun-exposed skin. Ocular melanoma occurs most commonly in the uvea, and uveal melanoma (UM) usually leads to unfavorable clinical outcomes and early metastasis (Shields, 2010; Krantz et al., 2017). Conjunctival melanoma (CM) is much rarer but also has a high degree of malignancy (Shields, 2000, 2010). The mortality rate following the initial diagnosis of UM is ∼30% at 5 years, but only 8% of people with UM survived 2 years with tumor metastasis (Shields, 2010; Krantz et al., 2017). The rate of survival for CM is ∼7% at 5 years (Shields, 2000). Due to the limitations in the treatment of these malignant tumors, there is an urgent need for a greater understanding of the role of circular molecules in melanoma especially in UM and CM.

In a study on CM, researchers screened out circMTUS1 (has_circ_0083444) from the highly expressed circRNAs in CM tissues compared with those of adjacent normal tissues (Shang et al., 2019). The host gene MTUS1 of circMTUS1 has been recognized as an antioncogene in multiple types of cancer, such as lung cancer (Gu et al., 2017b). In addition, it was found that circMTUS1 knockdown inhibited melanoma cell proliferation *in vitro* and suppressed tumor growth and the final weight of xenograft tumors in the mouse model (Shang et al., 2019). Furthermore, the bioinformatic analyses suggested that circMTUS1 may participate in CM progression through the ErbB, mitogen-activated protein kinase (MAPK), and Wnt signaling pathways and by targeting miR-1208 and miR-622 (Shang et al., 2019). The miR-622 is a proven tumor suppressor in cutaneous melanoma (Dietrich et al., 2018).

Yang et al. (2018) investigated dysregulated circRNA profiles in UM. Of studied circRNAs, upregulated circ_0128533 was predicted to work as a tumor promoter and protect UM cells from apoptosis by binding miR-145 whose overexpression could produce reverse effects (Li et al., 2014). Future studies should verify the function of these promising circRNAs.

In recent years, more and more circRNAs have been revealed to be involved in special characteristics of cancer, including resistance to cell death, limitless replication, sustained angiogenesis, tissue invasion, and metastasis (Su et al., 2019). Here, except for normal sponging miRNAs, circRNAs such as circ_0075804 can also perform oncogenesis regulation in cells by interacting with RBPs (Zhao et al., 2020). Listed studies reveal a novel scale of the role of circRNAs in ocular malignancy pathogenesis. Circ_0001649 and circ_0075804 may serve as diagnostic biomarkers, prognostic indicators for RB in the clinical works in the future as well as circMTUS1 for CM. These promising findings suggest that new treatment targeting these functional circRNAs may cure or suppress the formation of tumors in the eyes.

## Conclusion

In summary, through greater recognition of circular RNAs and powerful high-throughput sequencing combined with bioinformatic tools, more circRNAs are attracting the interest of researchers. CircRNAs play a vital role in regulating different molecules, signaling pathways, pathophysiological activities, and diseases. Herein, we depict the current landscape of the properties, biogenesis, and functions of circRNAs based on our understanding, and we introduce up-to-date advancements of circRNA studies in the scope of ocular diseases to help us focus on this rising star in research. The main finding is that dysregulation of specific circRNAs can act as potential biomarkers and make promising candidates for therapeutic intervention. However, except for circRNAs listed in Table 1, there are still many unknown circRNAs that can affect the development of eyes and the pathogenesis of ocular diseases (more than diseases that we discussed in this review), so more researches are urgently needed.

## Author Contributions

CZ and YY collected the information and drafted and revised the manuscript. JH contributed to collecting information and editing the manuscript. YY directed the work and finalized the manuscript. All authors read and approved the final manuscript.

## Conflict of Interest

The authors declare that the research was conducted in the absence of any commercial or financial relationships that could be construed as a potential conflict of interest.

## Abbreviations

ADAR1: adenosine deaminase acting on RNA 1
Ago: argonaute
AMD: age-related macular degeneration
ARCs: age-related cataracts
BAMBI: BMP and activin membrane bound inhibitor
CBS: cystathionine- β-synthase
CDK2: cyclin-dependent kinase 2
circRNA: circular RNA
ciRNAs: intronic circRNAs
CM: conjunctival melanoma
CNV: choroidal neovascularization
CRYAA: crystallin alpha A
CSPG4: chondroitin sulfate proteoglycan 4
DM: diabetes mellitus
DMNT3B: DNA (cytosine-5-)-methyltransferase 3 beta
DR: diabetic retinopathy
dsRNA: double-stranded RNA
E2F3: E2F transcription factor 3
ecircRNAs: exonic circRNAs
ECs: endothelial cells
EIciRNAs: exon-intron circRNAs
EMT: epithelial-mesenchymal transition
ERMs: epiretinal membranes
FVM: fibrovascular membranes
FVMs: fibrovascular membranes
FZD4: frizzled-4
HCEKs: corneal epithelial keratinocytes
Hcy: homocysteine
HG: high glucose
HHcy: hyperhomocysteinemia
HIPK3: homeodomain interacting protein kinase 3
HNRNPK: heterogeneous nuclear ribonucleoprotein K
HRMECs: human retinal microvascular endothelial cells
HRVECs: human retinal vascular endothelial cells
HuR: human antigen R
HUVECs: human umbilical vein endothelial cells
IOP: intraocular pressure
IRES: internal ribosome entry site
KIFAP3: kinesin-associated protein 3
lncRNAs: long noncoding RNAs
LOXL2: lysyl oxidase homolog 2
MBL: muscleblind
MEF2A: myocyte-specific enhancer factor 2A
METRN: meteorin
miRNAs: micro RNAs
MMP-2: matrix metallopeptidase 2
MREs: miRNA recognition elements
MTUS1: microtubule-associated scaffold protein 1
nAMD: neovascular age-related macular degeneration
ncRNAs: noncoding RNAs
NR3C1: nuclear receptor subfamily 3 group C member 1
nt: nucleotide
OIR: oxygen-induced retinopathy
PTEN: phosphatase and tensin homolog on chromosome ten
PVR: proliferative vitreoretinopathy
PWWP2A: PWWP domain containing 2A
QKI: quaking
RB: retinoblastoma
RBPs: RNA-binding proteins
RCMs: reverse complementary matches
RD: retinal detachment
RGCs: retinal ganglion cells
RISC: RNA-induced silencing complex
RNase R: ribonuclease R
ROP: retinopathy of prematurity
RPE: retinal pigment epithelium
RUNX2: runt-related transcription factor 2
SIRT1: sirtuin 1
SP1: specificity protein 1
ss: splice sites
STAT3: signal transducer and activator of transcription 3
UM: uveal melanoma
UTRs: untranslated regions
UV: ultraviolet radiation
VCAM1: vascular cell adhesion molecule 1
VEGF: vascular endothelial growth factor
WNT2: Wnt family member 2
XIAP: X-linked inhibitor of apoptosis
ZBTB44: zinc finger and BTB domain containing 44
ZNF: zinc finger protein
ZRANB1: zinc finger RAN-binding domain containing 1.
